# Preliminary Results of a New Auxiliary Mechatronic Near-Field Radar System to 3D Mammography for Early Detection of Breast Cancer †

**DOI:** 10.3390/s18020342

**Published:** 2018-01-25

**Authors:** Ashkan Ghanbarzadeh Dagheyan, Ali Molaei, Richard Obermeier, Andrew Westwood, Aida Martinez, Jose Angel Martinez Lorenzo

**Affiliations:** 1 Mechanical Engineering Department, Northeastern University, Boston, MA 02115, USA; ghanbarzadehdaghe.a@husky.neu.edu; 2 Electrical Engineering Department, Northeastern University, Boston, MA 02115, USA; molaei.a@husky.neu.edu (A.M.); obermeier.r@husky.neu.edu (R.O.); 3 Research Applications Specialist and Quantum Engineering Architect, Keysight Technologies, 65 Alsun Drive, Hollis, NH 03049, USA; andrew.westwood@keysight.com; 4 Harvard Vanguard, Wellesley, MA 02481, USA; aida_martinez@atriushealth.org

**Keywords:** bimodal breast imaging, breast cancer detection, Near-field Radar Imaging, antipodal Vivaldi antenna

## Abstract

Accurate and early detection of breast cancer is of high importance, as it is directly associated with the patients’ overall well-being during treatment and their chances of survival. Uncertainties in current breast imaging methods can potentially cause two main problems: (1) missing newly formed or small tumors; and (2) false alarms, which could be a source of stress for patients. A recent study at the Massachusetts General Hospital (MGH) indicates that using Digital Breast Tomosynthesis (DBT) can reduce the number of false alarms, when compared to conventional mammography. Despite the image quality enhancement DBT provides, the accurate detection of cancerous masses is still limited by low radiological contrast (about 1%) between the fibro-glandular tissue and affected tissue at X-ray frequencies. In a lower frequency region, at microwave frequencies, the contrast is comparatively higher (about 10%) between the aforementioned tissues; yet, microwave imaging suffers from low spatial resolution. This work reviews conventional X-ray breast imaging and describes the preliminary results of a novel near-field radar imaging mechatronic system (NRIMS) that can be fused with the DBT, in a co-registered fashion, to combine the advantages of both modalities. The NRIMS consists of two antipodal Vivaldi antennas, an XY positioner, and an ethanol container, all of which are particularly designed based on the DBT physical specifications. In this paper, the independent performance of the NRIMS is assessed by (1) imaging a bearing ball immersed in sunflower oil and (2) computing the heat Specific Absorption Rate (SAR) due to the electromagnetic power transmitted into the breast. The preliminary results demonstrate that the system is capable of generating images of the ball. Furthermore, the SAR results show that the system complies with the standards set for human trials. As a result, a configuration based on this design might be suitable for use in realistic clinical applications.

## 1. Introduction

Based on the most recent statistical report from the United States Cancer Statistics (USCS) database in 2013, breast cancer holds the highest rate of incidence among American women [1]. Moreover, the same study reports that after lung and bronchus cancer, breast cancer possesses the highest count of mortality among female patients. In spite of the significant 34% decrease in breast cancer death rates, from 1990 to 2010 [2,3], much effort is still needed for more accurate diagnosis and effective treatment. The frequency of false-positive results from mammography, with a reported prevalence of 9.6% [4], calls for the development of new techniques for cancer detection. Especially when false alarms could be a source of excessive short-term anxiety, and/or lead to multiple biopsies that some authors call unnecessary [5,6,7]. In the present paper, previous studies on breast cancer detection are collectively reviewed to address the necessity of enhancing the existing methods and devising novel techniques for more accurate detection of malignant tissues. This review is necessary, since it provides the engineering community a comprehensive description of the current methodologies for breast cancer screening. New findings suggest that multimodal screening systems, equipped with enhanced image quality and contrast, are capable of improving the accuracy of breast cancer detection. It is noteworthy to mention that prevention is another topic of interest that is predominantly overlooked despite its much lower inconvenience and cost for the patients. It has been estimated that 80% of all degenerative diseases, including cancer [8,9], and 30% to 50% of all cancer types [10] can be prevented with a comprehensive change in lifestyle and diet, thus reducing not only cancer mortality rates, but also incidence rates. This work only focuses on the main problems existing on current breast cancer detection systems, and it describe a set of potential solutions. Section 2, Section 3 and Section 4 are dedicated to reviewing the past and current perspectives on breast imaging. Section 5 presents the original idea of using microwave imaging as a complementary modality to 3D X-ray imaging in detail.

## 2. General Review on Mammography

In this section, the contributions of mammography to breast cancer detection and survival rates are discussed. Also, the shortcomings of this modality and its impact on patients are reviewed. Different approaches have been proposed to compensate for these limitations, two of which will be introduced in the next two sections.

### 2.1. Effect on Breast Cancer Survival Rate

The objective of regular screening is to find cancerous tissues or tumors before their symptoms begin to manifest, according to the American Cancer Society [11]. The effective reduction in breast cancer morbidity throughout the years is attributed to both earlier detection by mammography and progress in cancer treatment [2]. The role of combined mammography and adjuvant therapy in decreasing the rate of death from breast cancer was backed up in a 25-year study form 1975 to 2000 in the USA [12]. Specifically, adjuvant therapy was more likely to be efficacious when the cancer was detected at earlier stages. Effectiveness of mammography was also reported in studies in England and Wales (2000) and Dutch (2003) as a turning point in the trend of death rates; however, the role of therapy in these works was not distinguishable from that of mammography [13,14]. Four consecutive reports in 1997, 2000, 2001, and 2007 collectively demonstrated a positive association between mammography and morbidity reduction in Northern Sweden [15,16,17]. With further investigation and excluding the impact of therapy, a 10-year study in 2005 showed a 25% decrease in mortality, due to screening alone after the introduction of mammography in Copenhagen, Denmark [18]. Proponents of population-based mammography rely mostly on these promising results and are optimistic that, through regular screening, the chances of detecting tumors earlier would be higher. Annual mammography from the age of 40 is recommended for women by American Cancer Society, Society of Breast Imaging, and American College of Radiology, with the aim of detecting cancer in early stages [11,19]. Yet, World Health Organization (WHO) only recommends women to start regular screening from the age of 50–69 every two years [20]. Contrary to the articles just reviewed, a number of new studies have cast doubt on the effectiveness of mammography in its entirety due to its possible risks and disadvantages [21,22,23,24]. One critique on these new studies, which challenge the positive role of mammography, can be found in [25].

### 2.2. Main Drawbacks

**Figure 1:**
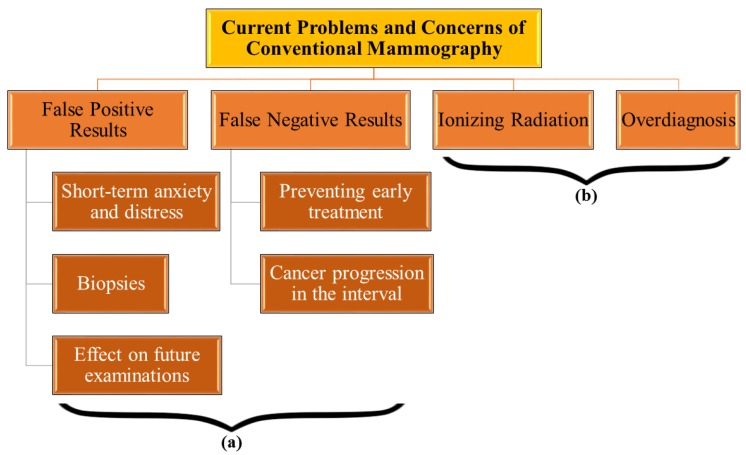
Current problems and concerns of mammography: (**a**) The problems that can be alleviated by the addition of a complementary imaging modality to mammography, (**b**) The concerns that are inherent to mammography and the addition of a complementary imaging system might not have a positive effect on them.

Though mammography has been able to enhance the possibility of early detection of breast cancer; false-positive (FP) and false-negative (FN) results, as well as over-diagnosis have raised some scientifically substantiated concerns (Figure 1). A research study in New England [26] showed a probability of 6.5% of false alarms among all investigated mammograms. This study reported that 23.8% of the participants received at least one false alert, and that the risk of getting an FP could be as high as 43.1% after nine screening sessions [26]. In 2005, Castell and colleagues’ study demonstrated a 10.6% prevalence of false alarm results in women (age range: 50–51) entering a screening program, which was reduced to 3.8% in the second and following sessions. The cumulative risk of the FP results was estimated to be 32.4% in their report, implying that one out of three women in Spain could receive a false alert during 10 biennial mammography [27]. A recent study in 2011 reported that there is a 16.3% possibility of false alarm on the first mammography and 9.6% at the succeeding sessions in the USA [4]. The cumulative probability for a woman to experience an FP, starting at age of 40 (50), was 61.3% (61.3%) and 41.6% (42.0%) with annual and biannual screening, respectively. A patient who is recalled for further assessment must undergo common procedures such as needle biopsy, ultrasound imaging, or perhaps surgical biopsy, before the chance of malignancy is eliminated. Several factors could lead to an FP reading, such as age, family history, number of previous biopsies, current usage of estrogen, and one of the most influential, radiologists’ tendency to interpret a mammogram as abnormal to avoid misdiagnosis [4,27]. The FN probability was estimated to be about 21% in film-screen mammography compared with 16% in computer-aided detection [28]. An earlier study in 1996 reported that between 12.5% and 31.5% of mammograms resulted in FN outcomes, depending on age, follow-up duration, and family history [29]. Current research is mostly concentrated on enhancing the radiologists’ diagnostic threshold, by introducing new methods to make image readings more confident and accurate. 

### 2.3. Effects of False-Positive Results

Main consequences of the FP results have been reported as a noticeable increment in women’s short-term anxiety, an alteration in their intention for future screening, and a boost in “false-positive biopsies”—as H. Welch and J. Passow have labeled them [30,31,32]. A Finnish study (2000) reported elevated levels of distress and anxiety about having breast cancer, accompanied by recurrent and lingering breast self-examinations (BSE), in patients who received false alarms [33]. Another study in Sweden (2003) reported similar results regarding BSE [34]. Although Marcia L. and others’ study in 1999 didn’t show a significant alteration in women’s general screening behavior [35], a recent 2014 study was in agreement with the previous findings on an increase in women’s short-term anxiety and their tendency to continue mammography [36]. McCann et al., reported a correlation between the risk of interval cancer (a cancer that starts between two successive mammograms) and the chance of FP results [37], and the reason why was speculated to be due to either hormone replacement therapy (HRT) or failure in the follow-up assessments that missed the tumor in the breast. In contrast to [36], McCann’s report demonstrated a decrease in patients’ intention for future attendance after getting an FP result.

In recent studies, FP results have been associated with an increased risk of developing breast cancer [38,39,40]. It was found that the risk is higher when an FP result is followed by biopsy than when only additional imaging is performed. Henderson et al. [38] speculated that the suspicious pattern that the radiologists detected in the first place, which turned out to be noncancerous, could be a marker of future cancerous activity and explain the extra risk. However, there are other factors that might contribute to the increased risk and need further research. These include tissue wounding [41,42,43,44], X-ray radiation for women who are carriers of BRCA1/2 mutations [45], and short- and long-term mental distress that is caused by the false results [32,33,46] and has been associated with higher risk of breast [47] and other types of cancers [48] as well as with adverse effects on the immune system [49,50,51,52].

Financial costs of all the sequential tests after an FP result, and the time lag after which one’s breast is declared cancer-free are also considerable. E. Lidbrink and colleagues estimated the entire cost of follow-up screenings and benign and surgical biopsies to be around £250,000 (about $394,000 in that time. Rates based on 1995 currency values, from [53]) in the first round of screening and £84,000 (about $132,000 in that time [53]) in the second round, per 1000 patients in 40–64 age span [54]. Moreover, it took almost 6 months for 73% of the patients in the first round and 64% of the patients in the second round to be declared free of cancer. For a minor portion of the studied population, this period lasted up to 22 months.

## 3. Digital Breast Tomosynthesis

### 3.1. Novel Approaches in DBT

Detecting tumors in dense breasts, which are those with 50% or more glandular tissue, is difficult using conventional X-ray systems. This needs to be addressed, since approximately 40% of women eligible for screening have a dense breast [55]. Digital Breast Tomosysthesis (DBT) utilizes full-field digital detectors and a rotating X-ray tube to scan the compressed breast [25,56]. The DBT, also known as 3D mammography, produces a 3D reconstruction of the breast tissue, enabling radiologists to extract and inspect any 2D layer of the breast at any given depth. Several algorithms such as Filtered Back Projection (FBP), Gaussian frequency blending (GFB), Maximum Likelihood Expectation Maximization Method (MLEM), and Simultaneous Algebraic Reconstruction Technique (SART) have been used to generate the 3D DBT reconstruction [57].

### 3.2. DBT Contributions to Breast Cancer Detection

Conventional mammography uses screen-film, with suitable resolution to detect micro-calcifications; however, its narrow dynamic range makes tumor visualization and detection difficult in dense regions of the breast [56]. On the other hand, full-field digital mammography (FFDM) utilizes detectors with wider dynamic range. Moreover, it provides two other advantages over screen-film mammography: the ability to manipulate images after screening and enhanced contrast between dense and fatty tissues. The accuracy of digital mammography was shown to be higher than that of the film-based one, particularly in women under the age of 50, patients with uniformly or extremely dense breasts, and premenopausal/perimenopausal women [58]. The DBT images of an FDA-approved breast phantom provided superior visibility of margins, lesions, and calcifications when compared to conventional mammography [59]. The DBT also provided better delineation of (1) boundaries of tumors, and (2) vessels around micro-calcifications, thus contributing to a better differentiation between benign and malignant tumors; and it used similar or less radiation exposure [59]. Teertstra et al. [60] also reported improved lesion detection using the DBT, and they suggested tomosynthesis as an additional screening system to conventional mammography for better examination of the breast. In the clinical setting, the combination of 2D and 3D mammography is preferred to and more commonly used than the DBT alone [56]. Bernardi et al. [61] reported an improved accuracy in breast imaging with combined 2D/3D mammography, which could lead to fewer FP results, and in turn, fewer follow-up biopsies. In accordance with this observation, Gur and his colleagues [62] found an average of 10% reduction in false alerts when a 2D/3D setup was used. Furthermore, in the recent 2014 study, Houssami et al. [63] reported an increase in the rate of true-positive results and higher sensitivity using the DBT as an additional diagnostic tool to the 2D mammography. In Bernardi’s subsequent 2014 study [64], meaningful improvement was found in radiologists’ true-positive evaluation—from 60% in 2D to 87% in 2D/3D. Moreover, 7% increase in sensitivity for dense breasts and 9% increase in specificity for all study groups were the results of 2D screening plus the DBT in TOMMY trial in the UK [65]. Based on these studies, it is concluded that the DBT has enhanced breast imaging performance; still, there is much room for progress in minimizing the FP and FN results. It should be noted that the publication bias and the funding bias were not investigated in the reviewed studies concerning the DBT.

## 4. Microwave Breast Imaging

Inasmuch as more contrast exists between the cancerous and fibro-glandular tissues in microwave frequencies [66], microwave imaging is a suitable candidate for further investigations as an addition to 2D/3D mammography. In microwave imaging of the breast, the retrieval of dielectric properties—permittivity and conductivity, whose values differ relatively largely between the aforementioned tissues—is the primary goal. In this regard, another modality of interest is millimeter-wave imaging that can combine high resolution with high contrast and has been recently investigated for breast cancer detection [67,68]. This study focuses on microwave imaging in the near field and introduces a potential solution (Section 5) to alleviate the limited resolution problem and other challenges that exist at microwave frequencies. An extensive review of breast microwave imaging systems by Nikolova, from the early works to the recent advances, can be found in [69]. In this section, a brief overview of microwave imaging is presented and some initial studies concerning its applications are reviewed.

### 4.1. Background

Imaging in the microwave spectrum is of particular interest since the permittivity and conductivity values between healthy and cancerous mammary tissues differ largely in this frequency band. In the large-scale study by Lazebnik et al. [70], it was found that the electrical properties discrepancy between normal tissues and malignancies can be as high as 10:1 for adipose-dominant, and as low as 10% for fibro-glandular-dominant regions in the breast. Though the latter appears to be low, it is still meaningfully higher than the contrast (1%) that has been observed in X-ray frequencies between the same types of tissues [66]. One should note that *in vivo* experiments have demonstrated larger permittivity values for healthy tissues than, and subsequently different contrast ratios from, those obtained in the previous *ex vivo* tests [71,72]. In other words, the contrast is shown to be lower in the *in vivo* experiments than that in the *ex vivo* ones; yet, it is still higher than the contrast that is X-ray frequencies.

In addition to improving the contrast between the aforementioned tissues, microwaves can feasibly penetrate the breast with its largely fatty content and limited volume [73]. Several articles in literature have been dedicated to develop practical algorithms for microwave imaging from years ago until recently. Iterative numerical methods based on Newton-Kantorovish, Levenberg-Marquardt, and conjugate gradient least square procedures [74,75,76]; finite element, finite difference time-domain [77,78,79], and finite difference frequency-domain approaches [80,81,82,83]; as well as ensemble empirical mode decomposition [84], to name a few, have been proposed for solving microwave imaging problems. There is a number of physical and technical challenges in microwave imaging approaches as well. Among these, limited resolution, tissue loss, motion and heterogeneity, and difficulties in forward modeling can be mentioned [69].

### 4.2. Breast Microwave Imaging at Experimental and Simulation Stage

Microwave imaging for breast cancer detection has been investigated in both far- and near-field configurations. Several recent studies have reported the feasibility of holographic far-field imaging (HFI) for clinical applications [85,86]. In 2013, Wang et al., presented the 2D images of a breast phantom, both in simulation and experiment, using the holographic far-field approach [86]. Later, they demonstrated the imaging results of 3D objects with high resolution using the same technique [87,88]. Paz et al., showed that a setup with random spatial distribution of the antennas performs better than a layout with uniform antenna distribution and avoids the generation of replicas in the image reconstructed by the HFI [89]. Near-field radar imaging (NRI) systems, due to their generally easy implementation in a limited space, have attracted more interest for breast cancer imaging during the last decades [90,91,92,93,94,95,96,97,98]. This study also focuses on a compact NRI system.

Among the early works in NRI, that of Meaney et al., in 2000 is a pioneer in clinical implementations [72]. They introduced a physical layout for microwave imaging from 0.3 to 1 GHz. Their clinical prototype involved a liquid container in which the breast could be in pendant position and microwave antennas illuminated the tissue through saline as a matching liquid. In this trial, human subjects were examined in sessions lasting 10–15 min for each breast, by a tomographic approach starting from the chest and ending at the nipple. The results had low resolution in the permittivity and conductivity images as expected, but they provided valuable insight into the potentiality of the NRI in practice. A later study from the same research group [83] reported a good agreement between images obtained from microwave imaging and the MRI on human subjects with negative mammograms (cancer-free) as well as phantoms. The images of layered phantoms of various shapes and sizes showed satisfactory contrast without using prior information, indicating the robustness of the imaging algorithm.

In terms of array design, many experimental and fabrication studies, similar to that of Meaney, Golnabi and others [83,99,100], used a cylindrical arrangement of the antennas that surrounded a target or a breast hanging freely in the coupling medium [101,102,103]. Klemm et al., however, introduced a hemispherical microwave antenna array to conform with the shape of the breast and presented the imaging results of different breast phantoms using delay-and-sum and data-adaptive beam-forming [104]. Further, they tested the system for low-contrast scenarios [105] and carried out small-scale clinical experiments [106]. The idea of conformal or wearable sensors for breast cancer imaging was also pursued by Bahramiabarghouei et al. [107], via designing a flexible antenna array, and Rahman et al. [97], via fabricating antennas using a flexible material. Other flexible sensor designs can be found in [87,108,109].

In addition, microwave imaging via space-time (MIST) beam-forming has been implemented in 2D and 3D layouts [110]. In this technique, an ultra-wideband (UWB) antenna array transmits the waves and subsequently a beamformer images the backscattered signal energy as a function of position. In lieu of reconstructing the whole map of breast through an inverse algorithm, the UWB radar only manifests regions of high backscattered energy levels, i.e., malignancies. Then, the data-adaptive algorithm effectively removes dominant backscatter in the skin-breast interface to minimize its overshadowing effect. Both 2D and 3D simulations on breast phantoms were encouraging and robust. More recent studies include those that employ microwave radar-based data as prior knowledge to microwave tomography [111], use NRI for axillary imaging as an aid to breast tumor detection [112], and monitor tumor growth status during neoadjuvant chemotherapy [113].

## 5. A Bimodal System: DBT Plus NRI

**Figure 2:**
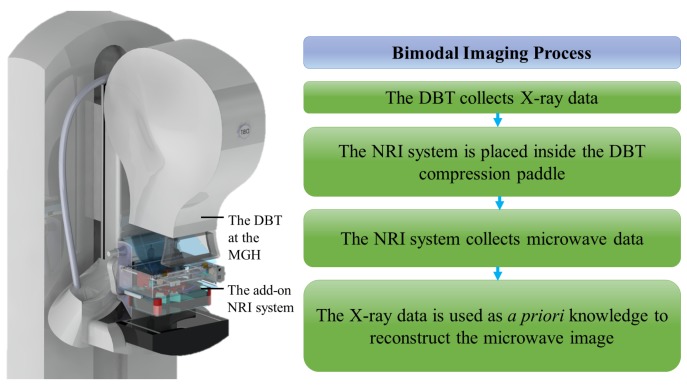
The bimodal imaging process using the DBT and NRI.

After reviewing the advantages and shortcomings of NRI and X-ray systems, it appears that a dual operating system of microwave and X-ray imaging could leverage the advantages of each approach and simultaneously compensate for some of their individual drawbacks. Two recent studies have evaluated the applicability of this idea by software simulations using fat distribution maps from the DBT [114,115]. This paper is an extension of [116]; and, at hand, it proposes an ultra-wideband NRI mechatronic system (NRIMS) that serves as a complementary scanning machine to the DBT to result in a bimodal hybrid imaging system. The fundamental idea behind the system is as follows (Figure 2): firstly, the DBT scans the breast with X-ray independently and collects the data required to reconstruct the 3D image; sequentially, NRIMS scans the tissue under compression one more time with microwaves (1–3 GHz) in a planar motion over the breast; next, the DBT image is used as a priori information by the microwave imaging algorithm in order to estimate the heterogeneous healthy tissue distribution in the breast. Here, the preliminary results of the NRIMS working in a standalone mode are presented for the sake of assessing its independent performance in a simple experiment. In the proceeding subsections, each part of the NRIMS is described [117,118].

### 5.1. Antipodal Vivaldi Antennas

**Figure 3:**
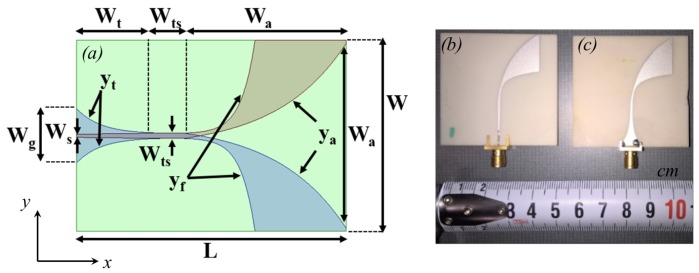
(**a**) The geometric design parameters of an AVA. The curves denoted by $y_{a}$, $y_{t}$, and $y_{f}$ are exponential. This transparent view of the antenna shows the curves on the front and back of the antenna (**b**) the fabricated antennas, signal side (left), and (**c**) ground side (right) [121].

Vivaldi antennas are compact, planar, and easy to fabricate [119]. Hence, they are a suitable candidate for an add-on NRI system. Nevertheless, these antennas, in general, require a balun to convert the microstrip into a strip-line and this eventually limits their bandwidth. One solution to overcome this restriction is to use Antipodal Vivaldi Antennas (AVAs), which have direct feeding microstrip lines and, at the same time, maintain the advantages of Vivaldi antennas. The AVAs presented in this work are designed and fabricated to radiate in a coupling medium instead of air, so that the electromagnetic energy coupled into the breast is enhanced. Using a coupling liquid with a high dielectric constant decreases each dimension of the antenna by a factor of $1/\sqrt{\epsilon_{r}}$, with $\epsilon_{r}$ being the relative permittivity of the liquid, which in turn enables the use of an array of antennas in a limited space. Such technique, however, requires a supportive substrate that has a high dielectric constant. A 2-mm ceramic substrate (T-Ceram, E-37) with a permittivity of about 37 meets this requirement. Figure 3 demonstrates the design parameters of an AVA as well as a photo of two fabricated AVAs. The parametric curves $y_{a}$, $y_{t}$, and $y_{f}$ shown in the figure are of exponential nature and follow the equations below [120]:

(1) $$y_{i}=\pm \left(A_{i}e^{P_{i}\left(x-B_{i}\right)}+C_{i}\right)$$

(2) $$C_{i}=\frac{y_{i1}e^{P_{i}x_{i2}}-y_{i2}e^{P_{i}x_{i1}}}{e^{P_{i}x_{i2}}-e^{P_{i}x_{i1}}}$$

(3) $$A_{i}=\frac{y_{i1}-y_{i2}}{e^{P_{i}\left(x_{i1}-B_{i}\right)}-e^{P_{i}\left(x_{i2}-B_{i}\right)}}$$

in which *i* can be substituted with *a*, *t*, or *f* to obtain $y_{a}$, $y_{t}$, or $y_{f}$, respectively. The constants $A_{i}$, $B_{i}$, $C_{i}$, and $P_{i}$ for each equation can be found in Table 1.

For the antennas built, the numerical values of the parameters given in Table 1 are as follows: W=30, $W_{g}=2.06$, $W_{a}=25.42$, $W_{ts}=0.29$, $W_{s}=0.025$, $L_{t}=8.40$, $L_{ts}=1.03$, $L_{a}=26.54$, all in millimeters; and $P_{t}=-1.04$, $P_{f}=0.94$, and $P_{a}=0.1$. More details about these antennas can be found in [122].

### 5.2. Selecting the Coupling Liquid

According to Lazbnik and colleagues’ comprehensive study [71], the dielectric constant of the breast can vary from as low as 4 to as high as 67 over 1–3 GHz, contingent upon its adipose content. In general, in healthy tissues, higher adipose percentage was associated with a lower averaged dielectric constant, and lower adipose content, or higher glandular and fibro-connective content, resulted in higher averaged dielectric constant. Rappaport [123] calculated the optimum dielectric constant for a presumed bolus liquid over 0.4 to 10 GHz, aimed at minimizing the reflection coefficient magnitude. The objective was to find the permittivity and conductivity values of a bolus liquid that enhanced the amount of energy coupled into the breast. It was shown that for 1.3, 2, and 3 GHz, the optimum relative permittivity was about 17.5, 32.5, and 65, respectively. Furthermore, as mentioned in the previous subsection, the dielectric constant of the selected ceramic was around 37. Considering these facts, and noticing that dielectric constant relaxation plots generally display a constant and then a decreasing trait as frequency goes up, the search for a suitable coupling liquid was limited to liquids whose dielectric constant were between 15 and 35 in 1.5–3 GHz range. The electrical properties of numerous liquids have been documented in the literature over a wide span of frequencies at 25 °C; hence, there were many cases to look into to find the desirable complex permittivity. As the first attempt, permittivity and conductivity behavior of binary aqueous mixtures of sodium chloride [124], ethanol [125], DMSO [126], DESO [127], glycine [128], ethylene glycol oligomer [129], 2-butoxyethanol [130], butyric acid [131], and 2-methoxyethanol [132] were reviewed. Later, other chemical compounds or their non-aqueous mixtures were also considered; among which methanol, 1-propanol, 2-propanol [133], dialkyl carbonates [134], 1-Hexanol/1-propenol [135], lithium salts/dimethyl carbonate [136], ethylene glycol - dimethyl sulfoxide [137], morpholine/n-butanol, and C16-ether-PN6-1ecith/methanol [131] can be named. Last efforts constituted reviewing a Ketone-like chemical, Dimethylketone (DMK), in mixture with 2–Butoxyethanol (2-BE) [138] and corn syrup [139]. Most liquids were ruled out based on the following criteria:

(i) The liquid must have a permittivity between that of the ceramic used as the antenna substrate and that of the breast ($\epsilon_{breast}<\epsilon_{liquid}<\epsilon_{ceramic}$).
(ii) The liquid must be non-toxic, non-carcinogenic, chemically stable, and possess relatively low viscosity and evaporation rate at the room temperature.
(iii) The AVAs must demonstrate acceptable performance in simulations using the selected liquid’s characteristics. The factors that need to be considered in the performance quality evaluation are the following: high directivity and a return loss above 10 dB.

**Figure 4:**
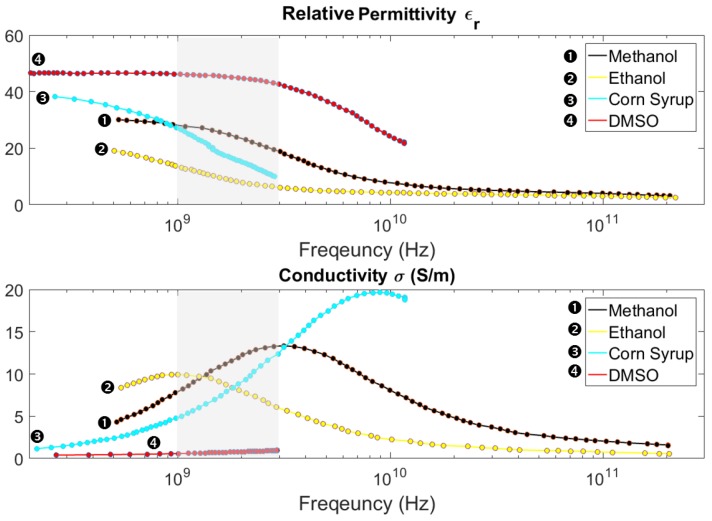
Relative permittivity and conductivity of four selected liquids. The frequency range of the system presented here (1–3 GHz) is shaded in gray. Data is taken from [125,126,133,139], severally.

Figure 4 illustrates the permittivity and conductivity plots for four different liquids as examples among those that were initially considered. These liquids met some of the criteria mentioned above, yet, eventually three of them were excluded due to high viscosity (corn syrup), higher dielectric constant than antennas’ substrate (DMSO), and possible carcinogenicity (methanol).

**Figure 5:**
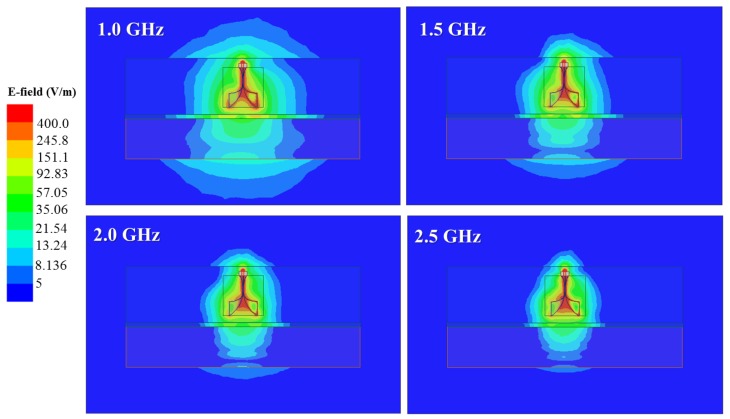
The absolute value of the electric field (E) induced by one AVA inside ethanol. The container and compression paddle are accounted for by a simplified model in HFSS. The top and bottom blocks represent the ethanol volume and the compressed breast, respectively. A thin layer of plastic (acrylic, 6 mm) is placed in between the blocks.

In the final analysis ethanol was selected, since it met almost all the conditions and was easily available for laboratory use. Previously, in [122] the performance of the designed AVA in a customized bolus liquid was presented. Here, Figure 5 illustrates how one AVA performs in ethanol, based on the liquid model given in [140] and using ANSYS HFSS software. It shows that the waves have penetrated the acrylic and polycarbonate plastic layer (bottom of the liquid container plus the paddle) into the breast. Also, the directivity of the AVAs in the 1–2.5 GHz frequency span is acceptable. In the test-bed presented in this work, only two antennas (transmitter/receiver) were built, for which the s-parameters and mutual coupling behavior inside ethanol can be found in [140].

### 5.3. Mechatronic System

**Figure 6:**
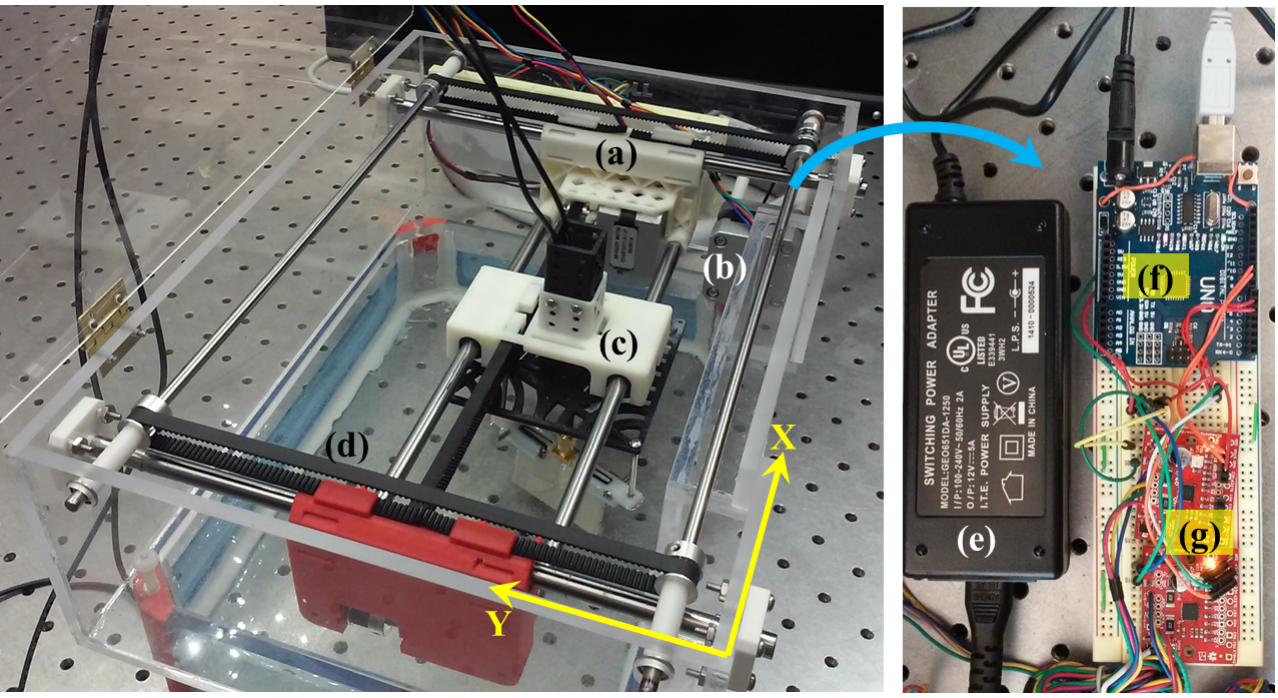
Mechatronic System: (**a**) Sliding motor and its holder, (**b**) fixed motor, (**c**) antenna holder, (**d**) belt-driven motion, (**e**) 12V/5A power supply for motors, (**f**) Arduino UNO, (**g**) Two Big Easy Drivers.

**Physical Layout.** For the test-bed, the antenna set needs to be moved in a predefined 2D path to collect the data over a horizontal plane. To achieve this purpose, a belt-driven mechanical layout was designed based on the open source 3D printer MAKERBOT Replicator. In this simple configuration, two stepper motors were mounted; one in a stationary position, and the other one on two steel bars, equipped with linear bearings, to make the planar motion possible. The fixed motor moved a secondary structure on the Y axis, including the other motor and the antenna set. The sliding motor moved the antennas on the X axis. The size and geometric qualities of the box containing all the mechanical parts were mainly restricted by *General Electric* DBT’s large compression paddle dimensions. Given these dimensions, two acrylic boxes were simply built, one on the top, holding the described XY actuator, and one on the bottom, serving as a liquid container. The bottom part was to fit into the paddle, and stand directly over the compressed breast area (Figure 6a–d).

An *Arduino UNO* board and two *Big Easy Drivers* were used to actuate the motors. A 12 V/5 A power supply was employed to feed the two motors with an average amperage of 0.6 A (each) under the load, and it was wired via Arduino power jack to the two driver’s power input (Figure 6f–g). To ensure that the box safely contains the coupling liquid, firstly, the bottom segment was sealed with a silicon-based sealant that was specifically designed for plastics (*General Electric*) and secondly, a lid with hinges was placed over the whole assemblage to constrain ethanol’s evaporation rate. Finally, small holes were made on the lid for the antennas’ cables and electronic wires to pass through.

**Figure 7:**
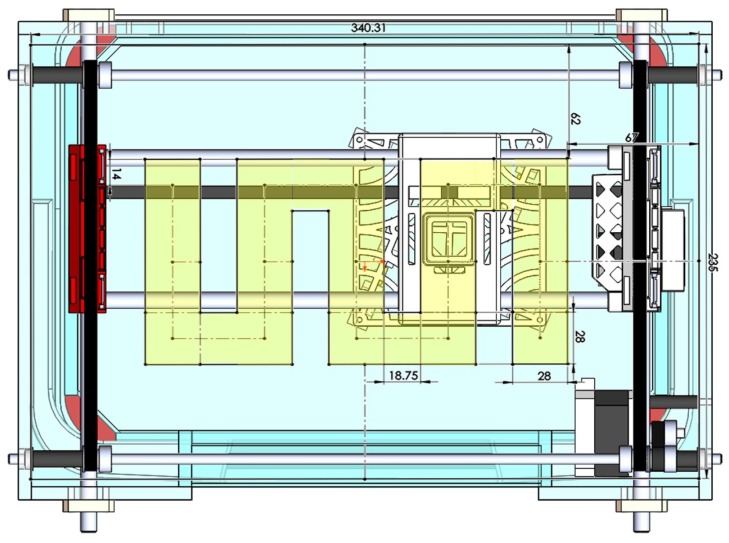
Schematic of the forward path the antenna set follows over the breast (the system top view, dimensions are in mm).

**Programming.** LabVIEW programming software was used to control the mechatronic system in sync with a vector network analyzer (KEYSIGHT, PNA-X N5242A). In order to establish the connection between Ardunio and LabVIEW, LabVIEW Interface for Arduino (LIFA) was installed on a Windows 10 workstation, and its already existing Stepper Motor Library was used to set the motion characteristics of the two motors. Motors were set to move at a constant speed of 2000 steps/s in each segment of the path, whose schematic is depicted in Figure 7. As can be observed, at any time in the motion, only one motor moves and the other one stays inactive.

Next, LabVIEW NI VISA MAX and Keysight IO Libraries Suite 17.1 were employed to configure the connection between the PNA-X and the PC, using a an Ethernet cable. Having both the PNA-X and two stepper motors connected to the host PC, a LabVIEW Virtual Instrument (VI) was developed to time the data acquisition and the mechanical motion. Since the motors and the control action from the drivers were of adequate precision, instead of using a position-sensor feedback solution, an open-loop one was adopted. Two approaches were possible to automate the system with that solution. One would be generating a Trigger Out signal from the PNA, after the s-parameter measurement on a specific position was done, and using it to command one of the motors to start moving to the next position. The other, and the less complex, method was to use time delays in order of milliseconds, one after the completion of motor motion (50 ms) and one after the data acquisition command (20 ms), to make sure each task was completed before moving forward to the next. Since both commands were executed relatively fast (70 ms delay in each step), the second method was selected. The overall travel and acquisition time was about 15 s. In the VI block diagram, the shape of the path, the separation between two consecutive steps on the path, and the number of frequencies at which parameters are evaluated ($N_{freq}$) can be modified.

The data was collected and recorded at each position by the PNA-X (Windows 7) on a folder shared over the network with the workstation PC. The data was stored in the s2p file format. The number “2” in the format name indicates a two-port measurement that constitutes the magnitude (dB) and phase (degrees) of {$S_{11},S_{12},S_{21},S_{22}$}, generating eight columns of data with 8 significant digits. In general, in an *N*-port measurement, the total count of complex parameters is $2N^{2}$ when the data format is magnitude/phase. A MATLAB script was used to remove the headings of the s-parameter files and assemble them together as one single data set in “CSV” format. This file was utilized for phase stability check and image reconstruction purposes. The data collection process could be ceased by an on/off switch on the power supply, in case there was software or physical failure.

### 5.4. The Bearing Ball Experiment

A simple experimental setup was designed to assess the imaging capability of the NRIMS in detecting a scatterer embedded inside a homogeneous medium. In the case of this experiment, for simplicity, the original path of the antennas was changed (Figure 7) into a straight line passing the center of the ethanol container, when viewed from the top. The system was configured to acquire 25 data sets in 25 equally-spaced positions along the path. The s-parameter data was sampled by 64 points over the frequency span in the calibration process resulting in an overall number of 8×64×25=1280 data in total.

**Figure 8:**
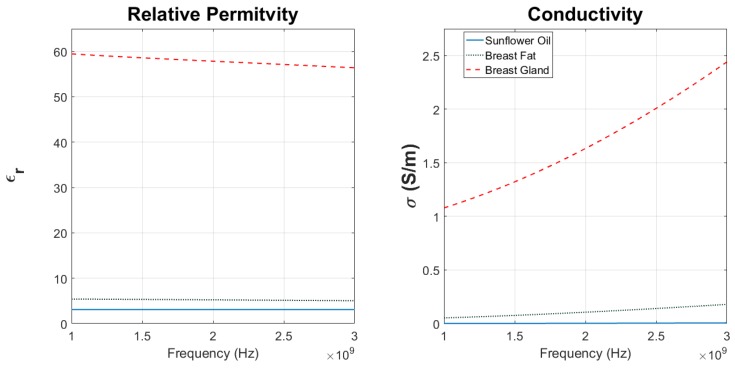
The relative permittivity $\epsilon_{r}$ (**left**) and conductivity σ (**right**) of sunflower oil (solid blue) are close to the behavior of breast fat (dotted green) for the selected frequencies when compared to that of other breast tissues such as fibro-glandular tissue (dashed red) [141,142].

Sunflower oil was selected as the background medium since its permittivity was relatively low, and it was comparable with that of human’s breast fatty tissue as shown in Figure 8. The mean value of relative permittivity for sunflower oil and breast fat over the 1–3 GHz span is 3.12 and 5.23, respectively [141,142]. Though their conductivity values are quite disparate (mean value of 0.003 S/m versus 0.1095 S/m in that frequency range, in order), sunflower oil seemed to be a good option for this initial test where the contrast parameter (the unknown in the imaging algorithm, Section 7) was defined based on relative permittivity and not conductivity. Another container was built for the sunflower oil in which the NRIMS could be partially immersed. The end was to eliminate any air gap between the ethanol container at the bottom of the system and the oil.

**Figure 9:**
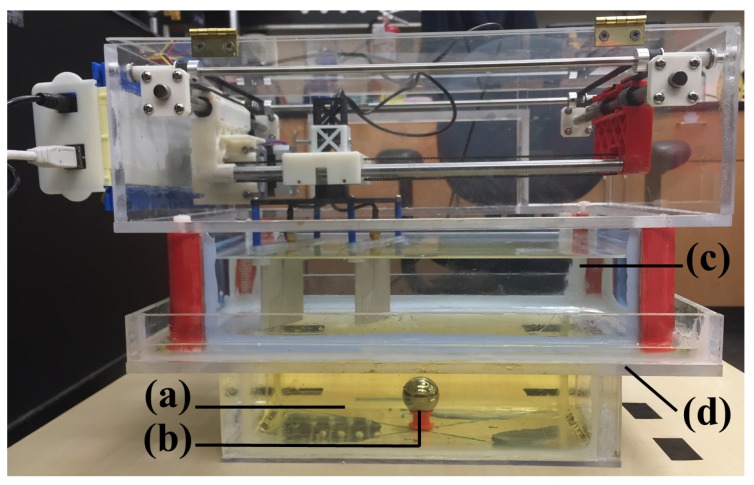
The bearing ball imaging experiment setup: (**a**) sunflower oil, (**b**) the bearing ball and its base, (**c**) the ethanol container, attached to the NRIMS and partially submerged in oil, (**d**) the oil container.

The target was a steel bearing ball mounted on a small plastic base and positioned in the center of the oil container, at the bottom. The size of the ball (1 inch in diameter) was chosen to be large compared to early-stage breast tumors, because the main goal of this study was to evaluate whether the system is able to detect an object in a uniform medium. Next, the experiment setup was assembled by putting the NRIMS over the oil container, centering the two containers from the top, connecting the antennas to the PNA, and finally linking the PC to the Arduino board. After loading the VI and powering the system, two measurements were carried out, one time without the ball, as the background, and one time with the ball. The background data was required for the imaging reconstruction process which is described in Section 7. In order to examine the robustness of the imaging algorithm, the ball was placed in different positions. In each measurement, the NRIMS needed to be lifted so that the ball could be either shifted or removed. Figure 9 shows a photo of the experiment layout when the ball was placed at the center.

**Figure 10:**
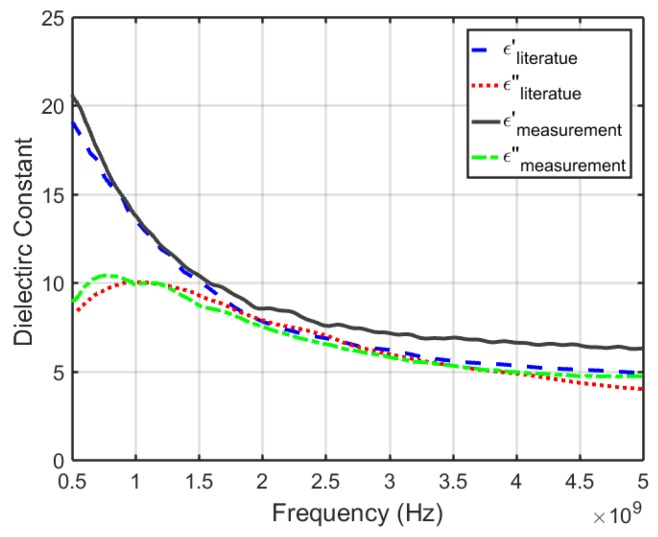
Dielectric properties of ethanol (200 proof) in the 0.5–5 GHz range; measured versus what has been reported in the literature [125]. $\epsilon^{\prime }$ and $\epsilon^{\prime \prime }$ denote the real and imaginary part of the complex relative permittivity, respectively.

For a more accurate model in the imaging algorithm and the SAR analysis, the dielectric properties of absolute ethanol (200 proof) were measured using the PNA material measurement software (Keysight Material Measurement Suite 2015) and then compared with the ones reported in [125]. The curves, as plotted in Figure 10, were in good agreement and the measured data did not affect the final results significantly.

## 6. The SAR Simulation

In order to ensure that the radiation from the antennas are safe for human subjects, a Specific Absorption Rate (SAR) simulation was carried out using one AVA as it radiated inside ethanol towards a breast model. SAR measures the amount of power a certain tissue absorbs when it is exposed to electromagnetic radiation [143]. The local SAR inside the tissue can be obtained as

(4) $$SAR_{local}\left(r,f\right)=\frac{{\sigma \left(r,f\right)|E\left(r,f\right)|}^{2}}{\rho (r)},$$

in which σ(r,f) is the material’s conductivity in [S/m], r is the position vector, ρ(r) is the mass density of the tissue at r in [Kg/m^3^], *f* is the frequency in [Hz], and E(r,f) is the electric field at position r and frequency *f* in [V/m]. For standardization purposes, this parameter is usually averaged over a small sample volume Δv(r) with a value of *V* [m^3^] as follows:

(5) $$SAR_{average}\left(r,f\right)=\frac{1}{V}\int_{\Delta v(r)}\frac{\sigma \left(r^{\prime },f\right){\left|E\left(r^{\prime },f\right)\right|}^{2}}{2\rho (r^{\prime })}dr^{\prime }$$

This energy flow per second reveals itself in the form of heat or temperature gradient over time inside the tissue and if it oversteps a certain value, it could cause harm. Federal Communications Commission (FCC) in the United States has set a threshold of 1.6 W/kg for peak SAR values in a 1-gram sample of head [143] or other body parts. In Europe, as stated by The Council of The European Union (CEU), the limit is 2 W/kg per 10-gram samples of body parts [144]. Furthermore, IEEE Standard 1528 provides a method for calculating the peak SAR in human head when it is exposed to radio frequency radiation, which can also be applied to other body part calculations [145].

## 7. Imaging Algorithm

In order to obtain the scattered field from the total and background fields measured during the data acquisition process, the following relationship can be used [115,146]:

(6) $$E_{s}=E_{t}-E_{b}\approx {\left(\nabla^{2}+k_{b}^{2}\right)}^{-1}k_{b}^{2}E_{t}\chi_{\epsilon }$$

where $E_{t}$ is the total field vector, $E_{b}$ is the background field vector, $k_{b}$ is the wave number in the background medium, and $\chi_{\epsilon }=\left(\epsilon_{t}-\epsilon_{b}\right)/\epsilon_{b}$ is the contrast between the total (unknown) and the background (known) media. All of these parameters are functions of the position vector r and the frequency *f*. Since $\chi_{\epsilon }$ is small in the case of breast cancer (about 10% as mentioned in Section 4), Born approximation can be applied and $E_{t}$ in Equation (6) can be replaced by the known field $E_{b}$. The linearized equation can then be expressed in the compressed form, y=Ax+η, in which $y\in C^{M}$ is the vector of measured scattered field, $x\in C^{N}$ is the contrast vector that is to be solved for, $A\in C^{M\times N}$ is the sensing matrix, and finally $\eta \in C^{M}$ is the measurement noise. In this case, the number of measurements is much less than the number of unknowns (M<N) and thereby a regularization method is needed to acquire a unique solution for x. Tikhonov regularization is a suitable technique for the problem at hand and gives an optimal solution to the convex optimization problem below:

(7) $$\frac{1}{2}||Ax-{y||}_{\ell_{2}}^{2}+{\lambda ||x||}_{\ell_{2}}^{2}$$

Subjectto:Re(diag(ϵb)x+ϵb)≥1Im(diag(ϵb)x+ϵb)≥0,

where λ is the regularization parameter, and the optimization conditions are to make the contrast parameter physically realizable ($\epsilon_{r}\geq 1,\sigma \geq 0$) [147]. In place of Equation (7), other regularization methods such as $\frac{1}{2}||Ax-{y||}_{\ell_{2}}^{2}+{\lambda ||x||}_{\ell_{1}}$ can be utilized with the same optimization conditions, as described in [115].

## 8. Results

### 8.1. SAR Analysis

**Figure 11:**
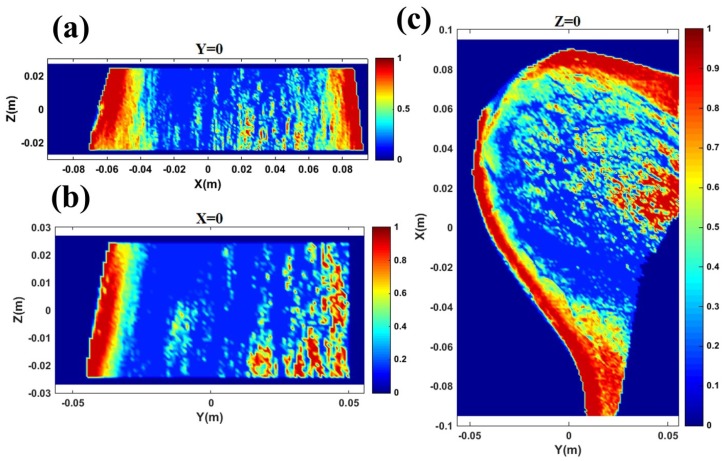
Fat distribution (dark red corresponds to 100% fat) in a healthy breast from a DBT image as seen from (**a**) front, (**b**) side, (**c**) and above. The margins of the breast are composed of 80–100% fat.

ANSYS HFSS was employed to study the SAR of the heat induced by an AVA inside a breast model placed under the antenna. The model constituted the data of fat distribution inside a real, healthy breast whose image was obtained by the DBT at the MGH. Figure 11 shows the fat distribution in the breast model from different views. The maximum value of 1 in the color bar shows 100% fat composition.

**Figure 12:**
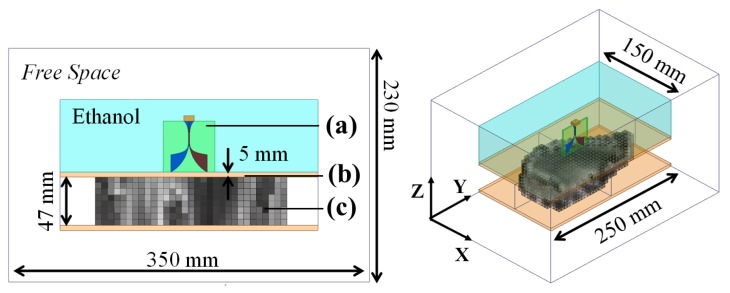
Simulation geometry in HFSS: (**a**) AVA, (**b**) acrylic sheet, (**c**) compressed breast model discretized into tiny cubes of 6 mm sides. On the right, an isometric view of the configuration is displayed.

The original model of the breast contained a large data matrix, so instead of importing the whole data set into HFSS, the fat ratio values were averaged in cubes of 6-mm sides and imported as a new object in the software, thus reducing the computational load. Note that the discretization for the full-wave simulation was still kept at one tenth of a wavelength inside each cube. Then, the complex dielectric properties of the breast were approximated with a Cole-to-Cole model. The effect of the compression paddle and ethanol container were also taken into account by setting up a simplified geometry of the real system as shown in Figure 12. In the real system, the input power by the PNA was 0 dBm (1 mW), and thus the simulations were configured accordingly.

**Figure 13:**
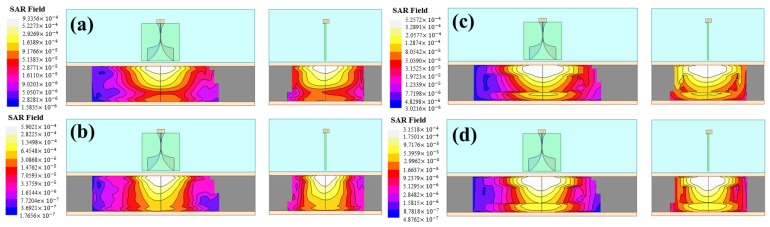
SAR analysis results with 1 g and 10 g samples at 1 GHz and 2.5 GHz: (**a**) 1 g at 1 GHz, (**b**) 1 g at 2.5 GHz, (**c**) 10g at 1 GHz, (**d**) 10 g at 2.5 GHz. The peak SAR is much smaller than the US (EU) standard, 1.6 W/Kg (2 W/Kg), in all settings.

HFSS automatically implements the IEEE Standard P1528.4 to compute the spatial-average of SAR inside 1- and 10-gram samples [148]. The results for both sample models in two different frequencies are displayed in Figure 13. It is apparent that the peak SAR in all the cases is certainly below the FCC and CEU standards, by at least three orders of magnitude, suggesting that the radiation from the antenna set is safe for human use, even if sixteen antennas were used instead of one.

### 8.2. Bearing Ball Imaging

**Figure 14:**
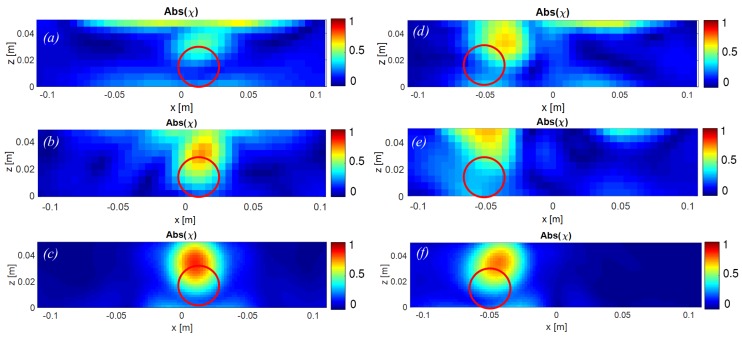
Reconstructed images for the contrast $\chi_{\epsilon }$ using (**a**) measurement; regular cable, ball located at x=0.01 m (**b**) measurement; phase-stable cable, ball located at x=0.01 m (**c**) simulation, ball located at x=0.01 m, (**d**) measurement; regular cable, ball located at x=−0.05 m, (**e**) measurement; phase-stable cable, ball located at x=0.01 m (**f**) simulation, ball located at x=−0.05 m. The values of the contrast are normalized with respect to the maximum value in each case.

Using the approach described in Section 7, the image of the ball was reconstructed in the XZ plane at two different positions: first at 1 cm off-center and second at 5 cm off-center, in the X direction. To assess the obtained results, the same geometry was also configured in MATLAB for both cases, and the problem was solved for the unknown variable $\chi_{\epsilon }$ using the Finite Differences in the Frequency Domain (FDFD) method. Figure 14 illustrates the results both from the simulations and the measurements. The measurements are carried out by both regular (a, d) and phase-stable (b, e) cables.

As can be observed, the retrieved images from the measurements show similar patterns to those obtained from simulations. They all present a maximum intensity signature at the surface of metallic ball, not inside of it where the electromagnetic fields are zero due to its high conductivity. Moreover, there are some artifacts in the images obtained from measured data, which are located at the interface between the mechatronic system and the sunflower oil. These artifacts suggest that there is a slight inaccuracy in the numerically computed Green’s Functions for the background media. Enhancing the accuracy of these functions, by measuring the radiated patterns, might improve the performance of the NRIMS even more.

The resolution of the images can be improved using a wider bandwidth, more data, and finer regularization. Further, case (b), with phase-stable cables, seems closer to the simulation than (a), which is done by regular cables. Yet, in case (e), the circular shape that appears in all other images has been lost. In other words, the phase-stable cables have not noticeably enhanced or degraded the quality or accuracy of the reconstructed images in comparison with the regular cables, in general. More scrutinization is needed to evaluate the effect of cable type on the final image quality.

## 9. Discussion

Using a simplified experimental layout, the developed NRI mechatronic system was assessed in terms of its capability to image a target. The experiment was designed only for proof-of-concept and accordingly it made the following simplifications: (i) the background medium (sunflower oil) was selected to be homogeneous, and (ii) the target was selected to be relatively large and a strong scatterer (a metallic ball, 1 inch in diameter). Though this experiment differs importantly from the case of the breast that has heterogeneous composition whose early-stage tumors are in order of millimeters, it shows the ability of the system to collect repeatable data from a medium and to translate it into dielectric maps or microwave images. In future, more realistic breast phantoms need to be imaged to show the full capability of the system. Also, the idea of using X-ray images as prior information for NRI, whose simulations results are presented in [115,147], can be materialized by collecting data from both the NRIMS and the DBT at the MGH. As shown by Jiang et al. in [103], in the case of a bimodal ultrasound-microwave system where ultrasound prior information was used for the NRI, it is predicted that using the prior information from the DBT could enhance the microwave image resolution significantly. In their case, the resolution was refined from 10 mm to 1.2 mm [103].

The novelty of the system designed, fabricated, and presented in this study lies in the fact that the NRIMS is compact, portable, and compatible with the DBT machine, enabling a co-registered scan of the breast. In other words, this system could be utilized as an add-on modality and scan the compressed breast within the same 3D mammography session, by being placed inside the compression paddle of the DBT. It should be noted that modifying the dimensions of the system’s frame allows it to be used in other state-of-the-art breast scanning machines such as automated breast ultrasound (ABUS) [149], molecular breast imaging (MBI) [150], and MicroDose SI [151]. In this regard, the NRIMS is unique from other microwave imaging systems, which require a separate scan session if they are to be used as an auxiliary modality [71,104,106,152]. Moreover, the NRIMS operates in the same setting of a mammography session with a compressed breast, eliminating the problem of tissue motion [69]. The problem of metallic parts interfering with magnetic fields in the MRI machine, which was reported by Golnabi [73] in the case of microwave-magnetic-resonance dual imaging, also does not arise in the NRIMS, due to using X-ray as the second modality and the ease in removing the NRIMS from the compression paddle.

## 10. Conclusions

This paper presents a comprehensive review of the literature associated with some of the major challenges existing in conventional breast imaging systems, particularly 2D mammography. This review acknowledged the need to improve current imaging systems, so that the breast cancer detection can be done at earlier stages of the disease while reducing false positive and negative rates. To the best of our knowledge, this paper presents the first NRI experimental system that can be used in a co-registered fashion with the DBT machine at the MGH. This system is equipped with a pair of UWB AVAs that are capable of radiating in a medium of high dielectric constant and can mechanically scan a medium using a LabView-Arduino virtual interface, developed in-house. The basic performance of the system was tested in order to produce the first set of images while keeping the radiated power levels safe for human exposure. The results show that, firstly, the Specific Absorption Rate (SAR) of the heat induced by the microwave radiation from the antennas in the tissue is indeed below the limit set by the FCC in the USA and the CEU in Europe; and, secondly, the system is capable of detecting a strong scattering object (a bearing ball) inside an oil container. The experimental images are in good agreement with those that were computationally generated by using an FDFD software. Notwithstanding that the preliminary results of this first prototype are recognizable, it is important to acknowledge that the experiment setup is rather simple in this work, since a comparatively large target was used. Therefore, the on-going research is aimed at examining how the NRIMS performs in detecting smaller inclusions in various and more complex geometries and background media of different material properties. Additionally, the use of the DBT images for prior information in the microwave reconstruction is currently under investigation, so that the fully hybrid, bimodal system could be used in its full potential in the clinical setting.

## Figures and Tables

**Table 1:** The parameters in the curve equations of the AVA.

*i*	$y_{i}\left(x\right)$	$A_{i}$	$B_{i}$	$C_{i}$	$P_{i}$
*t*	$y_{t}$	$\frac{W_{ts}-W_{g}}{2(e^{P_{t}L_{t}}-1)}$	0	$\frac{W_{g}}{2}-A_{t}$	$P_{t}$
*f*	$y_{f}$	$A_{f}$	$W_{t}+W_{ts}$	$\frac{W_{ts}}{2}-A_{f}$	$P_{f}$
*a*	$y_{a}$	$\frac{W_{ts}+W_{a}}{2(e^{P_{a}L_{a}}-1)}$	$W_{t}+W_{ts}$	$-\frac{W_{ts}}{2}-A_{f}$	$P_{a}$

## Abbreviations

The following abbreviations are used in this manuscript (in the order of appearance):

MGH	Massachuestts General Hospital
DBT	Digital Breat Tomosynthesis
NRI	Near-field Radar Imaging
SAR	Specific Absoprtion Rate
FP	False-positive
FN	False-negative
BSE	Breast self-examination
FFDM	Full-field Digital Mammography
UWB	Ultra-wideband
NRIMS	Near-field Radar Imaging Mechatronic System
AVA	Antipodal Vivaldi Antenna
PNA	Programmable Network Analyzer
FCC	Federal Communications Commission
CEU	The Council of European Union
